# A Simple Method for the Prediction of Human Concentration–Time Profiles and Pharmacokinetics of Antibody–Drug Conjugates (ADC) from Rats or Monkeys

**DOI:** 10.3390/antib11020042

**Published:** 2022-06-14

**Authors:** Iftekhar Mahmood

**Affiliations:** Mahmood Clinical Pharmacology Consultancy, LLC., Rockville, MD 20850, USA; iftekharmahmood@aol.com

**Keywords:** ADCs, human concentration–time profiles, pharmacokinetics, fixed exponent scaling

## Abstract

Knowledge of human concentration–time profiles from animal data can be useful during early drug development. The objective of this study is to predict human concentration–time profiles of antibody–drug conjugates (ADCs) and subsequently predict pharmacokinetic parameters in humans from rats or monkeys. Eight methods with different exponents of volume of distribution (0.8–1) as well as exponents of clearance (0.85), along with the exponents of volume of distribution for 5 ADCs, were used to predict human concentration–time profiles. The PK parameters were also scaled to humans from monkeys or rats using fixed exponents and compared with the PK parameters predicted from predicted human concentration–time profiles. The results of the study indicated that the exponent 0.9 and the combination of exponents of 0.9 and 0.8 (two exponents, 0.8 and 0.9, were used) were the best method to predict human concentration–time profiles and, subsequently, human PK parameters. The predicted PK parameters from fixed exponents were comparable with the predicted PK parameters estimated from human concentration–time profiles. The proposed methods are applicable to rats or monkeys with the same degree of accuracy. Overall, the proposed methods are robust, accurate, and cost- and time-effective.

## 1. Introduction

Antibody–drug conjugates (ADCs) are therapeutic products where a monoclonal antibody is linked to a pharmacologically active drug (a small molecule), forming a conjugate [1]. Initially, ADCs were developed for the treatment of oncology and hematology. The goal of an ADC as a cancer agent is to release the cytotoxic drug to kill tumor cells without causing any harm to healthy cells. It is now realized that besides oncology and hematology, ADCs can also be developed to treat other diseases such as inflammatory diseases, atherosclerosis, and bacteremia [2].

An ADC consists of three components: a monoclonal antibody, a cytotoxic drug (a small molecule drug, also known as the payload), and a linker that connects the antibody with the small molecule [3].

Following the administration of an ADC, several analytes can be found in the systemic circulation and measured by the available analytical methods. These analytes are the total antibodies (conjugated and un-conjugated antibodies), the conjugated antibody, the ADC (antibody with drug), and the un-conjugated drug (small molecule drug not conjugated to an antibody) [3].

A comparison of the pharmacokinetic profile (PK) of total antibodies with the PK of conjugated antibodies provides information on the rate of drug loss from the ADC. A wider difference in the concentration–time profiles (or exposure) between the total and conjugated antibodies indicates a more rapid loss of drug from the ADC. Sometimes, the concentrations of total and conjugated antibodies are indistinguishable. Un-conjugated antibodies are generally low in concentration and, sometimes, cannot even be measured at therapeutic doses of ADCs [3].

Interspecies allometric scaling is widely used to predict human PK parameters (clearance and volume of distribution) from animal data for both small and large molecules [4]. The current trend is to use a single species, mainly monkeys, to predict human PK parameters. It is widely believed that the monkey is the most suitable species to predict human PK parameters. This belief has led to the lack of research on other widely used species, such as rats or mice, for antibodies and ADCs. In two previous studies, Mahmood showed that a reasonable prediction of human clearance could be obtained from rats or mice [5,6].

Besides predicting human clearance and volume of distribution from animal data, it is also useful to predict the human concentration–time profile of a drug. Several manuscripts have used different methods to provide evidence that it is possible to predict the human concentration–time profile of a drug from animals [7,8,9,10,11,12]. A single-species scaling using monkeys has provided reasonably accurate predictions of human PK parameters for ADCs and monoclonal antibodies [5,6,13,14,15,16]. Mahmood demonstrated that rats can also be a useful species to predict human PK parameters, with almost the same degree of accuracy as monkeys for monoclonal antibodies and ADCs [5,6]. Li et al. [13], Deng et al. [14], and Oitate et al. [15] proposed a simple method termed as the ‘species-invariant time’ method to predict human concentration–time profiles from monkeys. The method is simple and fairly accurate. The method, however, can be improved for more accurate predictions of human concentration–time profiles and can be extended beyond monkeys to animals such as rodents.

The objective of this study is to propose and evaluate the predictive performance of different methods to predict human concentration–time profiles of ADCs based on rat or monkey concentration–time data.

## 2. Methods

A literature search was conducted to find rat, monkey, and human PK data for ADCs. Five ADCs were used in this analysis. The following is the description of the ADCs and the species and analytes used in the analysis. The analytes were measured either in the serum or plasma of ADCs. The dose selected for the extrapolation was in the linear range within the species. Where possible, the lowest animal or human dose within the linear range was selected for extrapolation and comparison.

1. Anti-NaPi2b (Lifastuzumab Vedotin DNIB0600A): Data are available from rats and monkeys but only for total antibodies [17,18].
2. Anti-5T4: Data are available from monkeys for three analytes (ADC, total, and payload) and were used to project human concentration–time profiles for all three analytes [19,20].
3. Trastuzumab deruxtecan: Data are available from monkeys for trastuzumab deruxtecan (DS-8201) and DXD. Total antibody and DS-8201 concentrations were similar in humans and animals. Therefore, total and DXD concentration–time profiles were projected in humans [21,22].
4. DSTA4637S: Data are available from rats and monkeys; total, conjugated, and un-conjugated analytes were used for concentration–time profile projections in humans [23,24].
5. Trastuzumab-DM1: Data are available from rats and monkeys for trastuzumab-DM1 (T-DM1) and total antibodies and were used for concentration–time profile projections in humans [25,26].

Mean concentration–time profiles for rats, monkeys, and humans were extracted using webplotdigitizer version 4.5 (copyright 2010–2021, Ankit Rohtagi). Generally, data were extracted reasonably accurately before day 1 (one or two data points) and accurately from day 1 onwards. An un-conjugated analyte was available for only one ADC (DSTA4637S).

### 2.1. Prediction of Human Concentration–Time Profiles from Rats or Monkeys

Eight methods were used for the prediction of concentration–time profiles in humans from rats or monkeys.

#### 2.1.1. Methods I–III

In these methods, the following equation with different exponents was used.

Predicted human concentration = Concentration at a given time in the animal × (dose in humans/dose in animals) × (weight of the animal/weight of human)^b^ (1)

Body weights for rats, monkeys, and humans used in this study were 0.25, 3.5, and 70 kg, respectively. The exponent ‘b’ was 1.0, or 0.90, or 0.80 on the ratio of animal to human body weight (the reason for choosing different exponents on the body weight is described in the Section 4).

#### 2.1.2. Methods IV–VI

Predicted human concentration = Concentration at a given time in the animal × (dose in humans/dose in animals)^0.85^ × (weight of the animal/weight of human)^b^ (2)

A modified version of methods I–III was to use a combination of exponents. Exponent 0.85 on the dose is an exponent of clearance for monoclonal antibodies. The exponent ‘b’ was 1.0 or 0.90 or 0.80.

#### 2.1.3. Methods VII–VIII

These two methods were developed to improve the prediction of concentration–time profiles in humans at the terminal phase. The previously described six methods underpredicted the concentrations of ADCs from day 14 onwards. These methods were used to improve the terminal phase of the concentration–time profile. Methods VII and VIII are based on the combination of two exponents, 1.0 and 0.8 or 0.9 and 0.8. For example, exponent 0.8 was used to predict human concentrations from day 14 onwards, but for all other time points before day 14, either exponent 1.0 or 0.9 was used.

The predicted human concentrations were compared with the observed human concentrations at the given time points. From the predicted concentration–time profiles in humans, PK parameters such as AUC, clearance, and half-life were predicted and compared with the observed human PK parameters. The observed human PK parameters were based on the concentration–time data digitally derived from the plots. This was done because the concentration–time profiles were based on the mean concentrations in the plots. The reported PK parameters were based on the mean PK values of several individuals. However, it should be noted that very little difference in the PK parameters was noted between the mean of individual PK parameters and the PK parameters derived from the mean concentration–time profile.

#### 2.1.4. Prediction of Human Clearance and Half-Life from Monkeys or Rats (Scaling Based on a Single Exponent)

The following method was used to predict the human clearance and half-life of ADCs from a single species (rats or monkeys).

Predicted CL in humans = CL of the species × (70/weight of the species)^0.85^ (3)

where CL of the species is the clearance of rats or monkeys, 70 is the human body weight in kilograms (kg), and 0.85 is the scaling exponent for clearance from animals to humans.

Predicted half-life in humans = Half-life of the species × (70/weight of the species)^0.15^ (4)

where the half-life of the species is the half-life of rats or monkeys, 70 is the human body weight in kg, and 0.15 (1–0.85) is the scaling exponent for half-life from animals to humans. Exponent 0.15 was obtained from the exponents of volume of distribution (1.0) and clearance (0.85) (discussed in the Section 4).

#### 2.1.5. Statistical Analysis

Predicted-to-observed concentration ratios by each method were calculated as follows:

Ratio = Predicted concentration/Observed concentration(5)

The number of concentrations by each method within 0.5–2, 0.5–1.5, 0.7–1.3, >2, and <0.5 fold errors was calculated for comparison across the methods.

Average fold error (AFE), which is the log-transformed ratio of the predicted and observed concentration values, was also reported for each method. For AFE, a value of 1.0 indicates no prediction error; AFE was calculated as follows:

AFE = 10^1/N∑log(concentration predicted/concentration observed)^ (6)

where AFE is the average fold error, N is the number of observations, and concentration_predicted_ and concentration_observed_ are the predicted and observed concentration values, respectively.

PK parameters from all methods were estimated, and the number of parameters by each method within 0.5–2, 0.5–1.5, 0.7–1.3, >2, and <0.5 fold errors was calculated for comparison across the methods.

## 3. Results

The initial analysis was done with exponent 1.0 on body weight, but it was noted that exponent 1.0 generally underestimated the concentrations of ADCs but substantially underestimated the concentrations from day 14 onward. Exponents 0.8 and 0.9 improved the concentration prediction to over 1.0, but 0.8 provided a better prediction in the terminal phase than exponent 0.9. However, exponent 0.8 slightly overpredicted the concentrations in the early phase. To compensate for the overestimation of exponent 0.8 at the early phase (showing an improvement in the terminal phase), a combination of exponents 0.8 and 0.9 was used. Therefore, exponent 0.8 was used to predict human concentrations from day 14 and onwards in methods VII and VIII.

**Figure 1:**
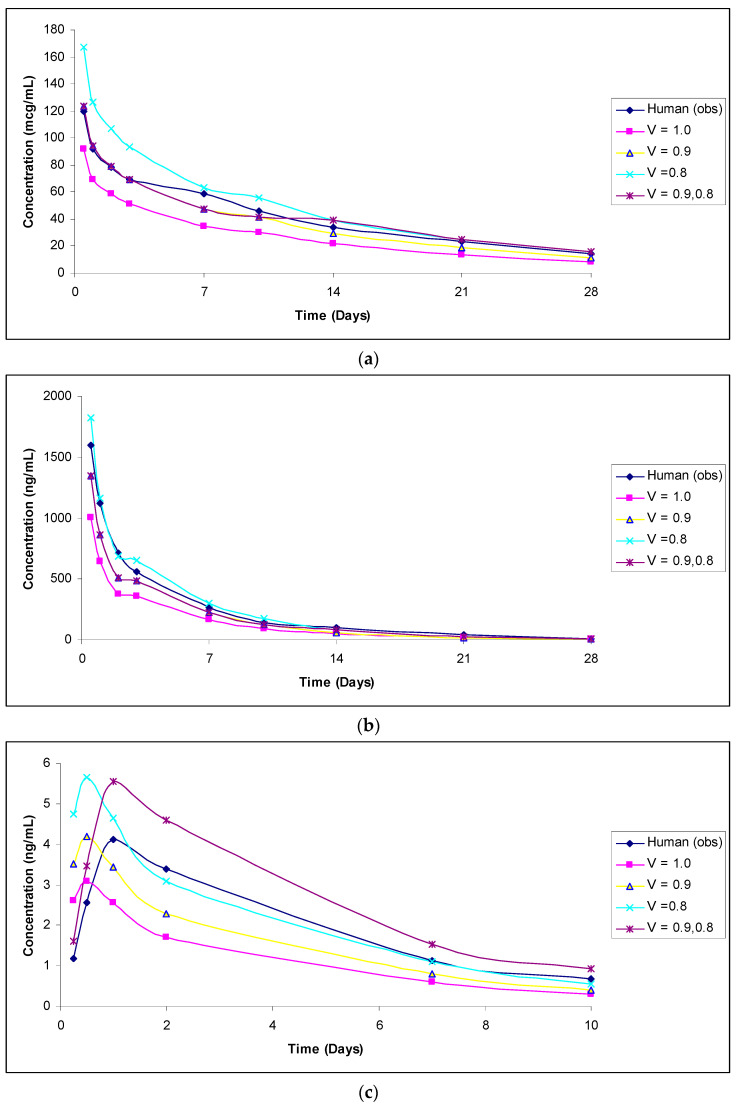
(**a**): Observed versus predicted plasma concentration–time profile of total DSTA4637S by exponents 1.0, 0.9, and 0.8, and 0.9 and 0.8 combined from 1 mg/kg monkey to 5 mg/kg human. (**b**): Observed versus predicted plasma concentration–time profile of conjugated DSTA4637S by exponents 1.0, 0.9, and 0.8, and 0.9 and 0.8 combined from 1 mg/kg monkey to 5 mg/kg human. (**c**): Observed versus predicted plasma concentration–time profile of un-conjugated DSTA4637S by exponents 1.0, 0.9, and 0.8, and 0.9 and 0.8 combined from 150 mg/kg monkey to 150 mg/kg human.

**Figure 2:**
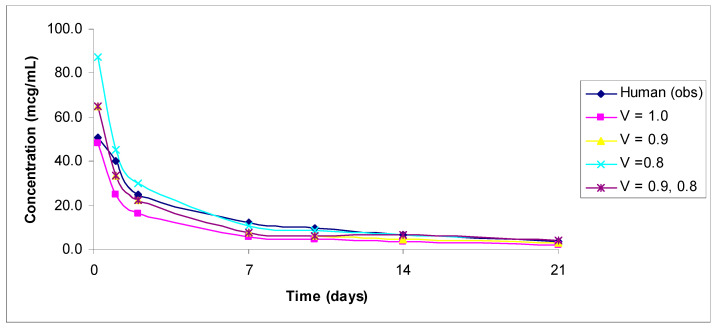
Observed versus predicted plasma concentration–time profile of total lifastuzumab vedotin by exponents 1.0, 0.9, and 0.8, and 0.9 and 0.8 combined from 1 mg/kg monkey to 2.4 mg/kg human. Only total anti-NaPi2b concentration–time data were reported by the authors [18].

**Figure 3:**
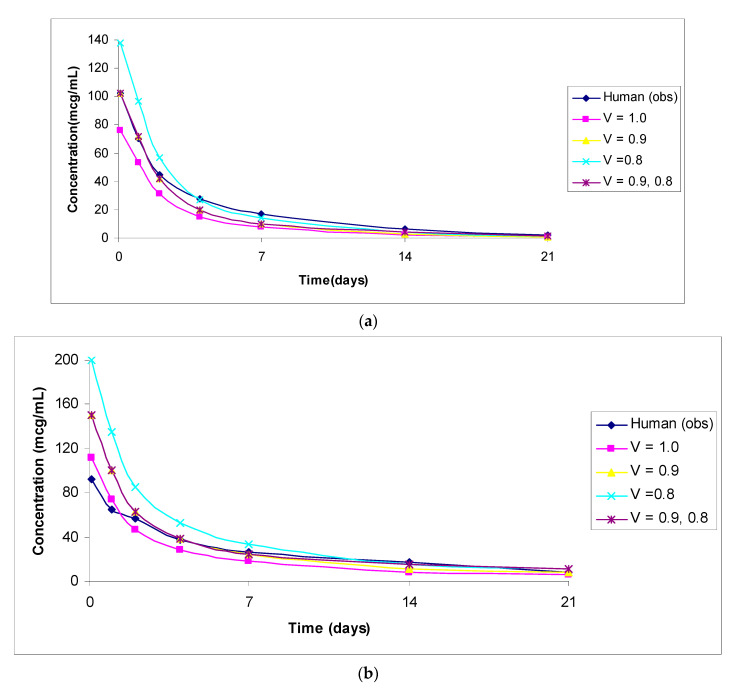

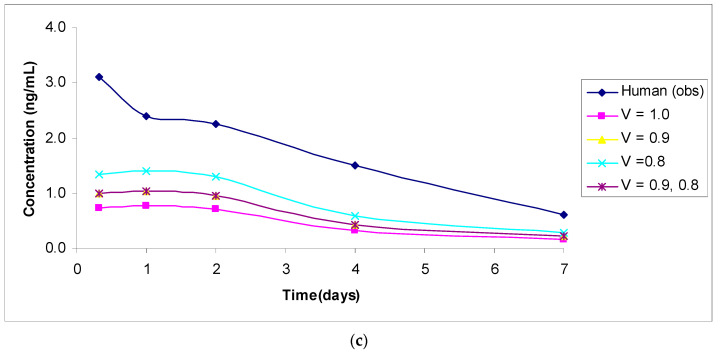
(**a**): Observed versus predicted plasma concentration–time profile of anti-5T4 (PF-06263507, 5-T4 ADC) by exponents 1.0, 0.9, and 0.8, and 0.9 and 0.8 combined from 3 mg/kg monkey to 3.4 mg/kg human. (**b**): Observed versus predicted plasma concentration–time profile of anti-5T4 (PF06281192, total antibody) by exponents 1.0, 0.9, and 0.8, and 0.9 and 0.8 combined from 3 mg/kg monkey to 3.4 mg/kg human. (**c**): Observed versus predicted plasma concentration–time profile of anti-5T4 (PF-06264490, payload) by exponents 1.0, 0.9, and 0.8, and 0.9 and 0.8 combined from 3 mg/kg monkey to 3.4 mg/kg human.

**Figure 4:**
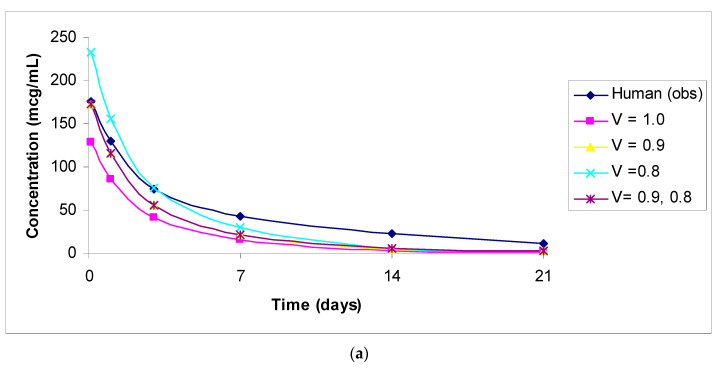

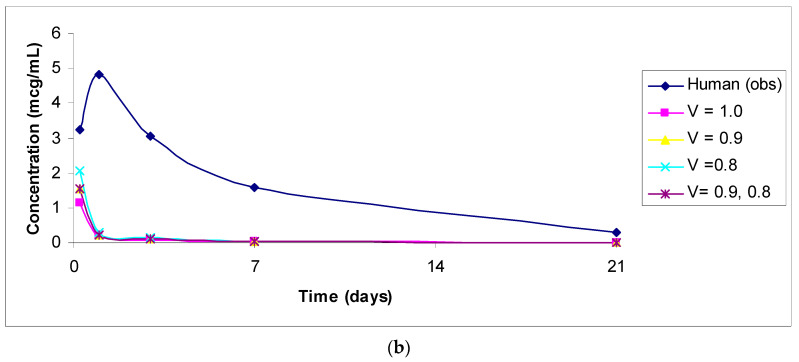
(**a**): Observed versus predicted plasma concentration–time profile of total trastuzumab deruxtecan by exponents 1.0, 0.9, and 0.8, and 0.9 and 0.8 combined from 3 mg/kg monkey to 6.4 mg/kg human. Total antibody and DS-8201 concentrations were similar in humans and animals. (**b**): Observed versus predicted plasma concentration–time profile of DXD by exponents 1.0, 0.9, and 0.8, and 0.9 and 0.8 combined from 8 mg/kg monkey to 6.4 mg/kg human.

**Figure 5:**
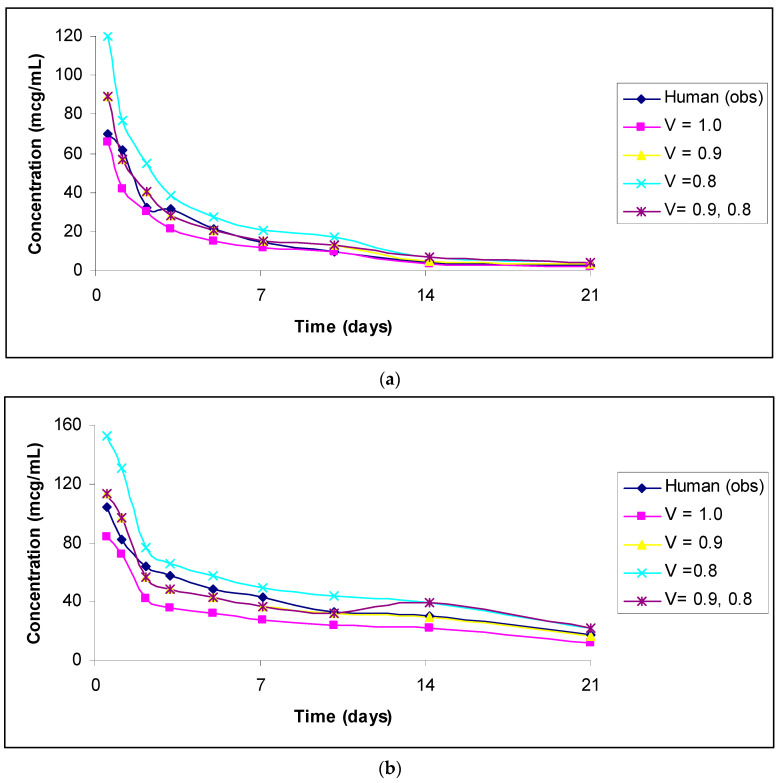
(**a**): Observed versus predicted plasma concentration–time profile of trastuzumab-DM1 (T-DM1) by exponents 1.0, 0.9, and 0.8, and 0.9 and 0.8 combined from 30 mg/kg monkey to 3.6 mg/kg human. DM1 animal data not available. (**b**): Observed versus predicted plasma concentration–time profile of total trastuzumab by exponents 1.0, 0.9, and 0.8, and 0.9 and 0.8 combined from 30 mg/kg monkey to 3.6 mg/kg human.

The predicted vs. observed human concentrations of analytes measured in serum or plasma are shown in Figure 1, Figure 2, Figure 3, Figure 4 and Figure 5. In the figures, the results for only the four most accurate methods are shown on a linear scale so that readers can clearly visualize the differences between the observed and predicted human concentrations.

In Table 1, projected human concentrations as a function of different ranges are shown. From monkey data, exponents 1.0, 0.9, and 0.8 and the combination of exponents 0.8 and 0.9 performed very well, but the remaining four methods mainly underpredicted (<0.5-fold prediction error) the concentrations. The number of concentrations falling within the 0.5 to 1.5 ratio was similar for all methods, with the exception of combination methods of CL (exponent 0.85) and exponents 1.0 or 0.9 for volume. These two methods were the worst methods for the prediction of human concentrations. When the range was narrowed (0.7–1.3) from 0.5 to 1.5, two methods (exponent 0.9 and combination of exponents 0.9 and 0.8) were the best among all methods for both total and conjugated antibodies (Table 1).

Out of 38 concentrations for total antibodies from monkeys, 30 and 32 concentrations were within the 0.7–1.3 range by exponent 0.9 and the combination of exponents 0.9 and 0.8, respectively (14 from exponent 1.0) (Table 1). Out of 25 concentrations for conjugated antibodies from monkeys, 18 concentrations were within the 0.7–1.3 range by exponent 0.9 and the combination of exponents 0.9 and 0.8 (9 from exponent 1.0) (Table 1). No ratio of the predicted-to-observed concentrations exceeded a 2-fold prediction error for any method.

From rats, the prediction of human concentrations of total and conjugated antibodies was poor from exponent 1.0 and other methods compared with exponent 0.9, exponent 0.8, and the combination of exponents 0.9 and 0.8. Most concentrations were underpredicted when exponent 1.0 was used, but the prediction was substantially improved when exponent 0.9 or the combination of exponents 0.8 and 0.9 was used. Out of 25 concentrations for total antibodies from rats, 10, 15, and 11 concentrations were within the 0.7–1.3 range by exponent 0.9, exponent 0.8, and the combination of exponents 0.9 and 0.8, respectively (1 from exponent 1.0). Out of 17 concentrations for conjugated antibodies from rats, 10, 3, and 9 concentrations were within the 0.7–1.3 range by exponent 0.9, exponent 0.8, and the combination of exponents 0.9 and 0.8, respectively (1 from exponent 1.0) (Table 1). No ratio of the predicted-to-observed concentrations exceeded a 2-fold prediction error, with the exception of two concentrations predicted from exponent 0.8. Like monkeys, the two best methods for rats were exponent 0.9 and the combination of exponents 0.9 and 0.8 (Table 1).

A clearer picture regarding the under- or overprediction of human concentrations from different methods emerges from the AFE comparison (Table 2). A systematic underprediction of human concentrations is noted for exponent 1.0 (<1.0 and, in some cases, far less than <1.0). Exponent 0.9 and the combination of exponents 0.8 and 0.9 provide the best prediction of concentrations, whereas exponent 0.8 appears to overpredict concentrations in many cases.

Overall, total and conjugated antibody concentrations were predicted fairly well by both rats and monkeys using exponent 0.9 and the combination of exponents 0.8 and 0.9.

The PK parameters of the five individual ADCs evaluated in this analysis are summarized in Tables S1–S7. Based on different ranges (Table S8) from the monkey data, the PK parameters of total and conjugated antibodies were predicted fairly accurately by exponent 0.9 or exponent 0.8 or the combination of exponents 0.8 and 0.9. A similar observation was noted for the rat data (Table S9).

Overall, the results of the study indicate that exponent 0.9 and the combination of exponents 0.8 and 0.9 provide the best results for concentration–time profiles and, subsequently, for the prediction of PK parameters for both total and conjugated drugs for rats or monkeys. Single-species scaling (rats or monkeys) using a fixed exponent of 0.85 for clearance and 0.15 for half-life provided accurate predictions for the clearance and half-life of ADCs using the concentration–time methods obtained from exponent 0.9 and the combination of exponents 0.9 and 0.8.

The concentration–time profile of only one un-conjugated antibody (DSTA4637S) was available and predicted from both rat and monkey data. The prediction of the conjugate of DSTA4637S was fairly good by exponent 0.9 and the combination of exponents 0.8 and 0.9 for both rats and monkeys. However, it should be noted that it is highly likely that the prediction of the concentrations of ADC conjugates will be erratic and unreliable. The reason for the erratic and unreliable prediction of un-conjugated ADCs is that the concentrations of un-conjugated ADCs are much lower (possibly not even detected reliably at therapeutic doses) than total and conjugated ADCs. Sometimes, it takes a much higher dose to detect the concentrations of un-conjugated ADCs. For example, for DSTA4637S, it was possible to project the concentrations of total and conjugated Abs from a 1 mg/kg monkey dose to a 5 mg/kg human dose. In contrast, for un-conjugated Abs, only two concentrations were detectable for a 15 mg/kg dose in monkeys and only seven concentrations from a 150 mg/kg dose in monkeys; these could be detected only till day 10.

## 4. Discussion

In this analysis, the evaluation of different methods to predict human concentration–time profiles for ADCs is an attempt to improve the predictive power of the proposed method by Oitate et al. for monoclonal antibodies, clarify the concept of the species-invariant time method, and show that rats are equally useful for the prediction of human concentration–time profiles for ADCs as monkeys.

A system is said to be ‘time invariant’ if the response of the system to an input is not a direct function of time [27]. In other words, an output signal does not depend on absolute time. This concept is used in system engineering for modeling purposes [27].

Pharmacokinetic space-time is a type of pharmacokinetic time where, when transformed to a species-dependent unit of chronological time, it utilizes an allometric variable (e.g., body weight, exponent of the allometry, etc.) for the estimation of PK parameters [28,29]. Chronological time, also known as species-invariant time, can be transformed into physiological time [29]. In chronological time, as the size of the animal increases, the heartbeat and respiratory rates decrease, but on the physiological time scale, regardless of their size, all mammals have the same number of heartbeats and breaths in their lifetime [28,29].

In PK, the concept of ‘species-invariant’ time was first applied by Dedrick [30] to methotrexate in five mammalian species following intravenous administration. By transforming chronological time to the equivalent time, the plasma concentrations of methotrexate were found to be superimposed in all species. Later, Boxenbaum [28,29] refined the concept of equivalent time by introducing two new units of pharmacokinetic time: kallynochrons and apolysichrons. Kallynochrons and apolysichrons are transformed time units in the elementary Dedrick plot and complex Dedrick plot, respectively. Over time, a lot of investigators have used species-invariant time method to predict plasma concentration–time profiles for many small molecule drugs [31,32,33,34]. The shortcomings of the species-invariant time method in the estimation of PK parameters and for the prediction of human concentration–time profiles are discussed by Mahmood and Yuan [8].

Species-invariant time is, in fact, a concept of superimposition, and, from both theoretical and practical perspectives, it requires several species. Although Oitate et al., Li et al., and Deng et al. termed their methods as species-invariant time based on a single species, the method is by no means the species-invariant time method. However, it should be recognized that whether or not the proposed methods by the aforementioned authors are termed species-invariant time methods, their proposed methods have provided a fairly accurate prediction of human concentration–time profiles and, subsequently, PK parameters.

In the current analysis, the concept was borrowed from the species-invariant time method in terms of application of volume of distribution, but the conversion of human equivalent time from monkeys or rats was ignored. Based on Oitate et al.’s exponent (clearance = 0.79 and 1.0 for volume), 1 day in cyno monkey time will be equal to 1.85 days (rounded to 2) in human time, and 1 day in rat time will be equal to 3.25 days (rounded to 3) of human equivalent time. In order to predict human concentration–time profiles from monkeys using the human equivalent time, the process is cumbersome, and many data points from monkeys may not be available. For example, for the prediction of human concentrations on days 7 and 21, monkey concentrations on days 3.5 and 10.5 will be needed, and it is unlikely that such time points will be available from monkeys. Similarly, in order to predict human concentration–time profiles from rats on days 7 and 28, rat concentrations on days 2.3 and 9.3 will be required, and concentrations at these time points will not be available. It is also true that the nearest available concentration and time can be used since the method is empirical and, at best, an approximation (although a fairly good approximation). Therefore, in this analysis, the conversion of time from rat or monkey time to human equivalent time was ignored, and actual time points and concentrations were used to predict the human concentration–time profile. This approach led to the underestimation of human concentrations (generally day 14 and onward) from exponent 1.0, but predictions were improved substantially by using exponent 0.9 or the combination of exponents 0.9 and 0.8.

In this study, three exponents (0.8, 0.9, and 1.0) for volume of distribution on body weight were used. In Equation (1), the exponent of 1.0 was used by the aforementioned authors on body weight, which is a widely used exponent for volume of distribution. It should be noted that exponent 1.0 is an allometric exponent for blood volume estimated from dozens of species, not for volume of distribution, as we know in PK [35]. It should be recognized that the allometric exponents are data-dependent (number of species, range of body weights, and the conditions under which a study is designed) and are not fixed in nature [35]. However, the use of exponent 1.0 for volume of distribution provides a reasonable prediction of the volume of distribution in humans from animals. When one uses a single species for scaling, there is no choice but to use a fixed number, which may and may not be appropriate for a given drug. Based on the allometric scaling of volume of distribution for both small and large molecules, the exponents of allometry were found to be widely variable. In the studies conducted by Mordenti et al. [36] and Mahmood [10] for macromolecules using at least three animal species, both authors noted allometric exponents ranging from 0.84–1.02 and 0.58 to 1.11, respectively (0.8 to 1.0 is the most common exponent range). Therefore, in this study, three exponents were used, and it was noted that the exponents provided different results with some practical applications. Exponent 1.0 slightly underestimated the concentrations of ADCs in the distribution phase but substantially underestimated the concentrations in the elimination phase (day 14 and onward). Exponent 0.9 improved the concentration prediction in both the distribution and elimination phases over exponent 1.0. On the other hand, exponent 0.8 overestimated the concentrations of ADCs in the distribution phase but considerably improved the concentration prediction in the elimination phase. These observations led to the conclusion that the best approach is either to use exponent 0.9 or the combination of exponents 0.9 and 0.8. Although, from the perspective of a 2-fold prediction error, exponent 1.0 provided fairly accurate predictions of human concentration–time profiles from the monkey data, the overall prediction can be substantially improved from exponent 0.9 or the combination of exponents 0.8 and 0.9. For rats, exponent 1.0 provided poor results and should not be used; for rats, the suitable exponents are exponent 0.9 or the combination of exponents 0.8 and 0.9.

Based on the allometric scaling of methotrexate, the exponent of half-life across five species was found to be 0.25 [35]. Since then, a notion has prevailed that the exponents of half-lives for drugs revolve around 0.25. This notion is not supported by other data [35]. In the species-invariant time method, the exponent of time is 0.25, derived from the difference between the exponent of volume of distribution (1.0) and the exponent of clearance (0.75). In this study, in order to predict half-life, exponent 0.15 was used, which is equal to (1–0.85), 0.85 being the exponent of clearance for macromolecules. Although allometrically and scientifically not robust, this approach provided a reasonable estimate of the half-lives of ADCs in humans. The predicted human PK parameters from rats or monkeys for the concentration–time profiles of ADCs and from one-species scaling with fixed exponents were comparable. The advantage of the current method over fixed exponent single-species scaling for the prediction of human PK parameters is that one can not only predict human PK parameters but will also get an idea about human concentration–time profiles, which can be useful.

## 5. Conclusions

The current proposed two methods (exponent 0.9 and the combination of exponents 0.9 and 0.8) for the prediction of human concentration–time profiles for total and conjugated antibodies are robust and accurate. The proposed methods from monkeys or rats are applicable with an equal magnitude of prediction accuracy. Although exponents 1.0 and 0.8 slightly underpredicted and overpredicted the human concentration–time profiles, these exponents can also be used for prediction purposes. By using exponents 1.0, 0.9, and 0.8, one can achieve the lowest and the highest prediction of human concentration–time profiles for total and conjugated antibodies, which will provide some insight into inter-subject variability. Although the combination of exponents 0.8 and 0.9 provides slightly better results than exponent 0.9 or exponent 0.8, the predicted half-life from this method may be slightly overestimated compared to exponent 1.0, and one should use caution if the predicted half-life from the combination of exponents 0.9 and 0.8 is substantially higher than the other exponents.

The predicted PK parameters from fixed exponents from rats or monkeys were comparable with the PK parameters estimated from predicted human concentration–time profiles. The half-life from the fixed exponent 0.15 may not always be accurate, but a more accurate estimate of half-life can be obtained from predicted human concentration–time profiles. Overall, the proposed method(s) to project human concentration–time profiles for total and conjugated antibodies is very simple and cost- and time-effective. It should be recognized that only five ADCs were used in this study, and only one set of data for un-conjugated ADCs was available from animals to predict concentration–time profiles in humans. The projected un-conjugated ADC in humans may be erratic and unreliable, as mentioned before.

## Figures and Tables

**Table 1:** Predicted versus observed concentration ratios within a certain range for different methods (concentration–time profile).

				V = 1.0	V = 0.9	V = 0.8	V = 1.0	V = 0.9
Range	V = 1.0	V = 0.9	V = 0.8	CL = 0.85	CL = 0.85	CL = 0.85	V = 0.8	V = 0.8
**Monkey (total) *n* = 38**								
<0.5	5	2	2	25	16	4	5	2
>2.0	0	0	0	0	0	0	0	0
0.5–1.5	33	34	32	13	22	34	33	35
0.7–1.3	14	30	22	3	12	14	20	32
**Monkey (conjugate) *n* = 25**								
<0.5	6	2	0	16	13	6	3	0
>2.0	0	0	0	0	0	0	0	0
0.5–1.5	19	23	20	9	12	16	20	20
0.7–1.3	9	18	14	6	9	7	10	18
**Rat (total) *n* = 25**								
<0.5	20	3	0	25	24	14	16	2
>2.0	0	0	2	0	0	0	0	0
0.5–1.5	5	22	20	0	1	8	6	23
0.7–1.3	1	10	15	0	0	3	4	11
**Rat (conjugate) *n* = 17**								
<0.5	8	2	1	17	15	8	8	1
>2.0	0	0	2	0	0	0	1	1
0.5–1.5	9	15	7	0	2	9	7	14
0.7–1.3	1	10	3	0	0	4	1	9

**Table 2:** Average fold error (AFE) values for different methods (concentration–time profile).

			V = 1.0	V = 0.9	V = 0.8	V = 1.0	V = 0.9
V = 1.0	V = 0.9	V = 0.8	CL = 0.85	CL = 0.85	CL = 0.85	V = 0.8	V = 0.8
**Lifastuzumab vedotin from monkey 1 mg/kg to 2.4 mg/kg human; Total antibody**							
0.54	0.73	0.99	0.30	0.41	0.55	0.65	0.80
**Lifastuzumab vedotin from rat 5 mg/kg to 2.4 mg/kg human**							
0.37	0.66	1.15	0.18	0.31	0.55	0.52	0.77
** PF-06263507 ** **from monkey 3 mg/kg to 3.34 mg/kg human**							
Total							
0.74	1.00	1.35	0.45	0.60	0.81	0.88	1.09
Conjugates							
0.55	0.74	0.99	0.33	0.44	0.60	0.65	0.80
Payload							
0.27	0.36	0.49	0.16	0.22	0.29	0.27	0.36
**Total trastuzumab deruxtecan from monkey 3 mg/kg to 6.4 mg/kg human**							
0.62	0.84	1.13	0.35	0.48	0.64	0.62	0.93
DXD							
0.04	0.06	0.07	0.03	0.04	0.05	0.04	0.06
**DSTA4637S from monkey 1 mg/kg to 5 mg/kg human**							
Total							
0.67	0.90	1.21	0.33	0.45	0.61	0.67	0.99
Conjugates							
0.54	0.72	0.98	0.27	0.36	0.49	0.54	0.80
Un-conjugated from monkey 150 mg/kg to 150 mg/kg human							
1.16	1.28	1.93	1.47	1.23	1.16	1.16	1.28
**V = 1.0**	**V = 0.9**	**V =0.8**	**CL = 0.85**	**CL = 0.85**	**CL = 0.85**	**V = 0.8**	**V = 0.8**
**DSTA4637S from rat 1 mg/kg to 5 mg/kg human**							
Total							
0.45	0.80	1.40	0.15	0.27	0.47	0.45	0.96
Conjugates							
0.32	0.56	0.99	0.11	0.19	0.33	0.32	0.65
Un-conjugated from rat 50 mg/kg to 150 mg/kg human							
0.58	1.03	1.80	0.21	0.37	0.66	0.58	1.03
** Trastuzumab-DM1 ** **from monkey 30 mg/kg to 3.6 mg/kg human**							
Total							
0.71	0.95	1.28	0.62	0.83	1.13	0.81	1.02
T-DM1							
0.83	1.13	1.52	0.73	0.99	1.33	0.95	1.20
** Trastuzumab-DM1 ** **from rat 3 mg/kg to 3.6 mg/kg human**							
Total							
0.32	0.57	1.00	0.14	0.24	0.42	0.42	0.65
TDM-1							
0.56	0.99	1.74	0.24	0.41	0.73	0.72	1.12

## Data Availability

All related data and methods are presented in this paper the Supplementary Materials. Additional inquiries should be addressed to the corresponding author.

## Supplementary Materials

The following supporting information can be downloaded at: https://www.mdpi.com/article/10.3390/antib11020042/s1, Table S1: Predicted and observed (human) pharmacokinetic parameters of Lifastuzumab vedotin from monkey and rat. Table S2: Predicted and observed (human) pharmacokinetic parameters of PF-06263507 (anti-5T4) from monkey. Table S3: Predicted and observed (human) pharmacokinetic parameters of trastuzumab deruxtecan. Table S4: Predicted and observed (human) pharmacokinetic parameters of DSTA4637S from rat. Table S5: Predicted and observed (human) pharmacokinetic parameters of DSTA4637S from monkey. Table S6: Predicted and observed (human) pharmacokinetic parameters of Trastuzumab-DM1 from rat. Table S7: Predicted and observed (human) pharmacokinetic parameters of Trastuzumab-DM1 from monkey. Table S8: Predicted versus observed PK parameters within different ranges for different methods for monkey. Table S9: Predicted versus observed PK parameters within different ranges for different methods for rat.
