# Supplementary material for: A Simple Method for the Prediction of Human Concentration–Time Profiles and Pharmacokinetics of Antibody–Drug Conjugates (ADC) from Rats or Monkeys
Source: Antibodies (Basel). 2022 Jun 14;11(2):42. doi: 10.3390/antib11020042 (PMC9219807; doi:10.3390/antib11020042)
Supplement: Supplementary file 1 [file antibodies-11-00042-s001.zip › antibodies-1643111-supplementary.pdf]

A Simple Method for the Prediction of Human Concentration-Time profiles and Pharmacokinetics of Antibody-Drug Conjugates (ADC) from Rat or Monkey

IFTEKHAR MAHMOOD, Ph. D.

Mahmood Clinical Pharmacology Consultancy, LLC.
e-mail: Iftekharmahmood@aol.com
Phone:301-838-4555

Table S1: Predicted and observed (human) pharmacokinetic parameters of Lifastuzumab vedotin from monkey and rat

Parameters	Human Observed	V = 1.0	V = 0.9	V =0.8	V = 1.0 CL = 0.85	V = 0.9 CL = 0.85	V = 0.8 CL = 0.85	V = 1.0 V = 0.8	V = 0.9 V = 0.8	1 species scaling
**From monkey 1 mg/kg to 2.4 mg/kg human; Total antibody**										
AUC	261	157	212	286	88	119	160	179	225	272
Ratio		0.60	0.81	1.10	0.34	0.45	0.61	0.69	0.86	1.05
Clearance	9.2	15.3	11.3	8.4	27.3	20.2	15.0	13.4	10.7	8.8
Ratio		1.66	1.23	0.91	2.97	2.20	1.63	1.45	1.16	0.96
Half-life	6.9	6.9	6.9	6.9	6.9	6.9	6.9	11.2	8.5	13.0
Ratio		1.00	1.00	1.00	1.00	1.00	1.00	1.62	1.23	1.88
**From rat 5 mg/kg to 2.4 mg/kg human; Total Antibody**										
AUC	261	104	182	320	50	87	153	146	209	348
Ratio		0.40	0.70	1.23	0.19	0.33	0.59	0.56	0.80	1.33
Clearance	9.2	23.1	13.2	7.5	48.3	27.5	15.6	16.5	11.5	6.9
Ratio		2.52	1.43	0.82	5.25	2.99	1.70	1.79	1.25	0.75
Half-life	6.9	7.9	7.9	7.9	7.9	7.9	7.9	48.4	13.6	23.1
Ratio		1.15	1.15	1.15	1.15	1.15	1.15	7.01	1.98	3.35

The units of AUC, CL, and half-life are µg*day/mL, mL/day/kg, and days, respectively.

The reported literature values for total antibodies were for AUC = 217 µg*day/mL, CL = 11.6 mL/day/kg; and half-life = 7.5 days, respectively.

Human observed values in the second column is based on digitally extracted human concentration-time data from the reported plots in the literature.

Table S2: Predicted and observed (human) pharmacokinetic parameters of PF-06263507 (anti-5T4) from monkey

Parameters	Human Observed	V = 1.0	V = 0.9	V =0.8	V = 1.0 CL = 0.85	V = 0.9 CL = 0.85	V = 0.8 CL = 0.85	V = 1.0 V = 0.8	V = 0.9 V = 0.8	1 species scaling
**From monkey 3 mg/kg to 4.34 mg/kg human; PF-06263507**										
AUC	404	229	309	416	138	186	251	416	320	442
Ratio		0.57	0.76	1.03	0.34	0.46	0.62	1.03	0.79	1.10
Clearance	10.8	19.0	14.1	10.4	31.4	23.3	17.3	10.4	13.6	9.8
Ratio		1.76	1.30	0.97	2.91	2.16	1.60	0.97	1.26	0.91
Half-life	4.6	4.0	4.0	4.0	4.0	4.0	4.0	4.0	4.5	6.4
Ratio		0.86	0.86	0.86	0.86	0.86	0.86	0.86	0.98	1.39
**From monkey 3 mg/kg to 4.34 mg/kg human; Total**										
AUC	646	506	682	921	305	412	556	742	793	835
Ratio		0.78	1.06	1.43	0.47	0.64	0.86	1.15	1.23	1.29
Clearance	6.7	8.6	6.4	4.7	14.2	10.5	7.8	5.8	5.5	5.2
Ratio		1.28	0.95	0.70	2.12	1.57	1.17	0.87	0.82	0.78
Half-life	7.3	7.8	7.8	7.8	7.8	7.8	7.8	14.5	10.1	9.4
Ratio		1.06	1.06	1.06	1.06	1.06	1.06	1.99	1.39	1.29
**From monkey 3 mg/kg to 4.34 mg/kg human; payload**										
AUC	12.9	3.7	5.0	6.7	2.2	3.0	4.0	3.7	5.0	NA
Ratio		0.28	0.38	0.52	0.17	0.23	0.31	0.28	0.38	NA
Half-life	2.6	3.0	3.0	3.0	3.0	3.0	3.0	3.0	3.0	
Ratio		1.15	1.15	1.15	1.15	1.15	1.15	1.15	1.15	

The units of AUC, CL, and half-life are µg*day/mL, mL/day/kg, and days, respectively.
The reported literature PK values for total antibody were for AUC = 418 µg*day/mL, CL = 10.0 mL/day/kg; and half-life = 6.0 days, respectively.

Only half-life of 8.8 and 3.4 days was given for total and payload, respectively.

Human observed values in the second column is based on digitally extracted human concentration-time data from the reported plots in the literature.

Table S3: Predicted and observed (human) pharmacokinetic parameters of trastuzumab deruxtecan

Parameters	Human Observed	V = 1.0	V = 0.9	V =0.8	V = 1.0 CL = 0.85	V = 0.9 CL = 0.85	V = 0.8 CL = 0.85	V = 1.0 V = 0.8	V = 0.9 V = 0.8	1 species scaling
**From monkey 3 mg/kg to 6.4 mg/kg human; trastuzumab deruxtecan**										
AUC	1047	436	588	793	248	335	452	436	610	719
Ratio		0.42	0.56	0.76	0.24	0.32	0.43	0.42	0.58	0.69
Clearance	6.1	14.69	10.89	8.07	25.80	19.12	14.17	14.69	10.49	8.9
Ratio		2.41	1.78	1.32	4.23	3.13	2.32	2.41	1.72	1.46
Half-life	7.2	4.3	4.3	4.3	4.3	4.3	4.3	4.3	5.0	6.1
Ratio		0.60	0.60	0.60	0.60	0.60	0.60	0.60	0.69	0.85
**From monkey 8 mg/kg to 6.4 mg/kg human; DXD**										
AUC	35.9	1.2	1.6	2.2	0.8	1.1	1.5	1.2	1.6	NA
Ratio		0.03	0.05	0.06	0.02	0.03	0.04	0.03	0.05	NA
Half-life	5.5	6.3	6.3	6.3	6.3	6.3	6.3	6.3	6.3	
Ratio		1.14	1.14	1.14	1.14	1.14	1.14	1.14	1.14	

The units of AUC, CL, and half-life are µg*day/mL, mL/day/kg, and days, respectively.

The reported literature values for trastuzumab deruxtecan were for AUC = 1030 µg*day/mL, CL = 6.4 mL/day/kg; and half-life = 7.3 days, respectively.

Human observed values in the second column is based on digitally extracted human concentration-time data from the reported plots in the literature.

Table S4: Predicted and observed (human) pharmacokinetic parameters of DSTA4637S from rat

Parameters	Human Observed	V = 1.0	V = 0.9	V =0.8	V = 1.0 CL = 0.85	V = 0.9 CL = 0.85	V = 0.8 CL = 0.85	V = 1.0 V = 0.8	V = 0.9 V = 0.8	1 species scaling
**From rat 1 mg/kg to 5 mg/kg human; Total Antibody**										
AUC	1349	581	1020	1792	196	344	605	581	1353	1112
Ratio		0.43	0.76	1.33	0.15	0.26	0.45	0.43	1.00	0.82
Clearance	3.7	8.6	4.9	2.8	25.5	14.5	8.3	8.6	3.7	4.5
Ratio		2.33	1.32	0.75	6.90	3.93	2.24	2.33	1.00	1.22
Half-life	12.9	12.2	12.2	12.2	12.2	12.2	12.2	12.2	12.2	15.8
Ratio		0.94	0.94	0.94	0.94	0.94	0.94	0.94	0.94	1.22
**From rat 1 mg/kg to 5 mg/kg human; Conjugate**										
AUC	5.8	2.1	3.7	6.5	0.7	1.3	2.2	2.1	3.9	7.1
Ratio		0.36	0.64	1.13	0.12	0.22	0.38	0.36	0.68	1.22
Clearance	12.3	33.8	19.2	10.9	100.1	57.0	32.4	33.8	18.2	10.0
Ratio		2.75	1.56	0.89	8.14	4.63	2.64	2.75	1.48	0.81
Half-life	4.7	3.5	3.5	3.5	3.5	3.5	3.5	3.5	4.6	6.1
Ratio		0.75	0.75	0.75	0.75	0.75	0.75	0.75	0.97	1.30
**From rat 50 mg/kg to 150 mg/kg human; unconjugated**										
AUC	21.3	5.9	10.4	18.2	2.2	3.8	6.6	5.9	10.4	NA
Ratio		0.28	0.49	0.86	0.10	0.18	0.31	0.28	0.49	NA
Half-life	3.2	1.5	1.5	1.5	1.5	1.5	1.5	1.5	1.5	
Ratio		0.48	0.48	0.48	0.48	0.48	0.48	0.48	0.48	

The units of AUC, CL, and half-life are µg*day/mL, mL/day/kg, and days, respectively. The reported literature PK values for total antibody were for AUC = 2060 µg*day/mL, CL = 2.7 mL/day/kg; and half-life = 19.2 days, respectively. For conjugate, PK values were for AUC = 5.9

μg*day/mL, CL = 11.1 mL/day/kg; and half-life = 5.3 days, respectively. For unconjugated, the AUC and half-life values were 23.1 ng*day/mL and 4.2 days, respectively.

Human observed values in the second column is based on digitally extracted human concentration-time data from the reported plots in the literature.

Table S5: Predicted and observed (human) pharmacokinetic parameters of DSTA4637S from monkey

Parameters	Human Observed	V = 1.0	V = 0.9	V =0.8	V = 1.0 CL = 0.85	V = 0.9 CL = 0.85	V = 0.8 CL = 0.85	V = 1.0 V = 0.8	V = 0.9 V = 0.8	1 species scaling
**From monkey 1 mg/kg to 5 mg/kg human; Total Antibody**										
AUC	1349	862	1163	1569	432	583	786	862	1391	1471
Ratio		0.64	0.86	1.16	0.32	0.43	0.58	0.64	1.03	1.09
Clearance	3.7	5.8	4.3	3.2	11.6	8.6	6.4	5.8	3.6	3.4
Ratio		1.57	1.16	0.86	3.13	2.32	1.72	1.57	0.97	0.92
Half-life	12.9	10.0	10.0	10.0	10.0	10.0	10.0	10.0	12.4	16.5
Ratio		0.78	0.78	0.78	0.78	0.78	0.78	0.78	0.96	1.28
**From monkey 1 mg/kg to 5 mg/kg human; Conjugate**										
AUC	5.8	3.4	4.6	6.1	1.7	2.3	3.1	3.4	4.8	7.4
Ratio		0.58	0.79	1.06	0.29	0.39	0.53	0.58	0.82	1.28
Clearance	12.3	21.2	15.7	11.6	42.2	31.3	23.2	21.2	14.9	9.6
Ratio		1.72	1.27	0.94	3.43	2.54	1.89	1.72	1.21	0.78
Half-life	4.7	4.1	4.1	4.1	4.1	4.1	4.1	4.1	4.4	6.3
Ratio		0.87	0.87	0.87	0.87	0.87	0.87	0.87	0.94	1.34
**From monkey 150 mg/kg to 150 mg/kg human; unconjugated**										
AUC	21.3	21.3	12.7	17.1	23.0	8.1	10.9	14.7	12.7	NA
Ratio			0.59	0.80	1.08	0.38	0.51	0.69	0.59	NA
Half-life	3.2	3.0	3.0	3.0	3.0	3.0	3.0	3.0	3.0	
Ratio		0.94	0.94	0.94	0.94	0.94	0.94	0.94	0.94	

The units of AUC, CL, and half-life are µg*day/mL, mL/day/kg, and days, respectively. The reported literature PK values for total antibody were for AUC = 2060 µg*day/mL, CL = 2.7 mL/day/kg; and half-life = 19.2 days, respectively. For conjugate, PK values were for AUC = 5.9

µg*day/mL, CL = 11.1 mL/day/kg; and half-life = 5.3 days, respectively. For unconjugated, the AUC and half-life values were 23.1 ng*day/mL and 4.2 days, respectively.

Human observed values in the second column is based on digitally extracted human concentration-time data from the reported plots in the literature.

Table S6: Predicted and observed (human) pharmacokinetic parameters of Trastuzumab-DM1 from rat

Parameters	Human Observed	V = 1.0	V = 0.9	V =0.8	V = 1.0 CL = 0.85	V = 0.9 CL = 0.85	V = 0.8 CL = 0.85	V = 1.0 V = 0.8	V = 0.9 V = 0.8	1 species scaling
**From rat 3 mg/kg to 3.6 mg/kg human; Total trastuzumab**										
AUC	1050	318	559	982	133	234	411	815	747	8780
Ratio		0.30	0.53	0.94	0.13	0.22	0.39	0.78	0.71	0.84
Clearance	3.4	11.3	6.4	3.7	27.1	15.4	8.8	4.4	4.8	4.1
Ratio		3.33	1.89	1.08	7.96	4.53	2.58	1.30	1.42	1.21
Half-life	10.8	9.6	9.6	9.6	9.6	9.6	9.6	18.3	10.0	20.5
Ratio		0.89	0.89	0.89	0.89	0.89	0.89	1.70	0.93	1.90
**From rat 3 mg/kg to 3.6 mg/kg human; trastuzumab-DM1 (TDM1)**										
AUC	293	163	286	503	68	120	210	206	314	456
Ratio		0.56	0.98	1.72	0.23	0.41	0.72	0.70	1.07	1.56
Clearance	12.3	22.1	12.6	7.2	52.9	30.1	17.1	17.5	11.5	7.9
Ratio		1.80	1.02	0.58	4.30	2.45	1.39	1.42	0.93	0.64
Half-life	4.8	5.5	5.5	5.5	5.5	5.5	5.5	6.7	5.2	11.1
Ratio		1.14	1.14	1.14	1.14	1.14	1.14	1.40	1.07	2.31

The units of AUC, CL, and half-life are µg*day/mL, mL/day/kg, and days, respectively. The reported literature PK values for total trastuzumab were for AUC = 1270 µg*day/mL, CL = 3.3 mL/day/kg; and half-life = 12.5 days, respectively.

For TDM1, PK values were AUC = 295 µg*day/mL, CL = 12.9 mL/day/kg; and half-life = 3.5 days, respectively.

Human observed values in the second column is based on digitally extracted human concentration-time data from the reported plots in the literature.

Table S7: Predicted and observed (human) pharmacokinetic parameters of Trastuzumab-DM1 from monkey

Parameters	Human Observed	V = 1.0	V = 0.9	V =0.8	V = 1.0 CL = 0.85	V = 0.9 CL = 0.85	V = 0.8 CL = 0.85	V = 1.0 V = 0.8	V = 0.9 V = 0.8	1 species scaling
**From monkey 30 mg/kg to 3.6 mg/kg human; Total trastuzumab**										
AUC	1050	642	866	1169	563	760	1025	1790	1168	1000
Ratio		0.61	0.83	1.11	0.54	0.72	0.98	1.70	1.11	0.95
Clearance	3.4	5.6	4.2	3.1	6.4	4.7	3.5	2.0	3.1	3.6
Ratio		1.65	1.22	0.91	1.88	1.39	1.03	0.59	0.91	1.06
Half-life	10.8	10.5	10.5	10.5	10.5	10.5	10.5	41.0	16.7	16.5
Ratio		0.97	0.97	0.97	0.97	0.97	0.97	3.80	1.54	1.53
**From monkey 30 mg/kg to 3.6 mg/kg human; trastuzumab-DM1 (TDM1)**										
AUC	293	236	319	430	207	279	377	259	332	474
Ratio		0.81	1.09	1.47	0.71	0.95	1.29	0.88	1.13	1.62
Clearance	12.3	15.2	11.3	8.4	17.4	12.9	9.5	13.9	10.9	7.6
Ratio		1.24	0.92	0.68	1.41	1.05	0.78	1.13	0.88	0.62
Half-life	4.8	5.8	5.8	5.8	5.8	5.8	5.8	9.7	6.2	8.2
Ratio		1.20	1.20	1.20	1.20	1.20	1.20	2.03	1.29	1.71

The units of AUC, CL, and half-life are µg*day/mL, mL/day/kg, and days, respectively. The reported literature PK values for total trastuzumab were for AUC = 1270 µg*day/mL, CL = 3.3 mL/day/kg; and half-life = 12.5 days, respectively.

For TDM1, PK values were AUC = 295 µg*day/mL, CL = 12.9 mL/day/kg; and half-life = 3.5 days, respectively.

Human observed values in the second column is based on digitally extracted human concentration-time data from the reported plots in the literature.

Table S8: Predicted versus observed PK parameters within different ranges for different methods for monkey

Range	V = 1.0	V = 0.9	V =0.8	V = 1.0<br>CL = 0.85	V = 0.9<br>CL = 0.85	V = 0.8<br>CL = 0.85	V = 1.0<br>V = 0.8	V = 0.9<br>V = 0.8	1 species<br>scaling
**AUC (total) n = 5**									
Ratios	0.60	0.81	1.10	0.34	0.45	0.61	0.69	0.86	1.05
	0.57	0.76	1.03	0.34	0.46	0.62	1.03	0.79	1.29
	0.42	0.56	0.76	0.24	0.32	0.43	0.42	0.58	0.69
	0.64	0.86	1.16	0.32	0.43	0.58	0.64	1.03	1.09
	0.61	0.83	1.11	0.54	0.72	0.98	1.70	1.11	0.95
<0.5	1	0	0	5	5	1	1	0	0
>2.0	0	0	0	0	0	0	0	0	0
0.5-1.5	4	5	5	0	0	4	4	5	5
0.7-1.3	0	4	5	0	0	1	1	4	4
**Clearance (total) n = 5**									
Ratios	1.66	1.23	0.91	2.97	2.20	1.63	1.45	1.16	0.96
	1.76	1.30	0.97	2.91	2.16	1.60	0.97	1.26	0.78
	2.41	1.78	1.32	4.23	3.13	2.32	2.41	1.72	1.46
	1.57	1.16	0.86	3.13	2.32	1.72	1.57	0.97	0.92
	1.65	1.22	0.91	1.88	1.39	1.03	0.59	0.91	1.06
<0.5	0	0	0	0	0	0	0	0	0
>2.0	1	0	0	0	0	0	1	0	0
0.5-1.5	0	4	5	0	1	1	3	4	5
0.7-1.3	0	4	4	0	0	1	1	4	4
**Half-life (total) n = 5**									
Ratios	1.00	1.00	1.00	1.00	1.00	1.00	1.62	1.23	1.88
	0.86	0.86	0.86	0.86	0.86	0.86	0.86	0.98	1.29
	0.60	0.60	0.60	0.60	0.60	0.60	0.60	0.69	0.85
	0.78	0.78	0.78	0.78	0.78	0.78	0.78	0.96	1.28
	0.97	0.97	0.97	0.97	0.97	0.97	3.80	1.54	1.53

Table S8 continued

Range	V = 1.0	V = 0.9	V =0.8	V = 1.0 CL = 0.85	V = 0.9 CL = 0.85	V = 0.8 CL = 0.85	V = 1.0 V = 0.8	V = 0.9 V = 0.8	1 species scaling
**Half-life (total) n = 5**									
<0.5	0	0	0	0	0	0	0	0	0
>2.0	0	0	0	0	0	0	1	0	0
0.5-1.5	5	5	5	5	5	5	4	4	3
0.7-1.3	4	4	4	4	4	4	2	3	3
**AUC (conjugate) n = 3**									
Ratios	0.78	1.06	1.43	0.47	0.64	0.86	1.15	1.23	1.1
	0.58	0.79	1.06	0.29	0.39	0.53	0.58	0.82	1.28
	0.81	1.09	1.47	0.71	0.95	1.29	0.88	1.13	1.62
<0.5	0	0	0	2	1	0	1	0	0
>2.0	0	0	0	0	0	0	0	0	0
0.5-1.5	3	3	3	1	2	2	3	3	2
0.7-1.3	2	3	1	1	1	2	2	3	2
**Clearance (conjugate) n = 3**									
Ratios	1.28	0.95	0.70	2.12	1.57	1.17	0.87	0.82	0.91
	1.72	1.28	0.95	3.44	2.55	1.89	1.72	1.22	0.78
	1.24	0.92	0.68	1.41	1.05	0.78	1.13	0.88	0.62
<0.5	0	0	0	0	0	0	0	0	0
>2.0	0	0	0	2	1	0	0	0	0
0.5-1.5	2	3	3	1	1	2	2	3	3
0.7-1.3	2	3	3	0	1	2	2	3	2

Table S8 continued

Range	V = 1.0	V = 0.9	V =0.8	V = 1.0<br>CL = 0.85	V = 0.9<br>CL = 0.85	V = 0.8<br>CL = 0.85	V = 1.0<br>V = 0.8	V = 0.9<br>V = 0.8	1 species<br>scaling
**Half-life (conjugate) n = 3**									
Ratios	1.06	1.06	1.06	1.06	1.06	1.06	1.99	1.39	1.29
	0.87	0.87	0.87	0.87	0.87	0.87	0.87	0.94	1.34
	1.20	1.20	1.20	1.20	1.20	1.20	2.03	1.29	1.71
<0.5	0	0	0	0	0	0	0	0	0
>2.0	0	0	0	0	0	0	1	0	0
0.5-1.5	3	3	3	3	3	3	1	3	2
0.7-1.3	3	3	3	3	3	3	1	2	1

Table S9: Predicted versus observed PK parameters within different ranges for different methods for rat

Range	V = 1.0	V = 0.9	V =0.8	V = 1.0 CL = 0.85	V = 0.9 CL = 0.85	V = 0.8 CL = 0.85	V = 1.0 V = 0.8	V = 0.9 V = 0.8	1 species scaling
**AUC (total) n = 3**									
Ratios	0.40	0.70	1.23	0.19	0.33	0.59	0.56	0.80	1.33
	0.43	0.76	1.33	0.15	0.26	0.45	0.43	1.00	0.82
	0.30	0.53	0.94	0.13	0.22	0.39	0.78	0.71	0.84
<0.5	3	0	0	0	0	0	1	0	0
>2.0	0	0	0	0	0	0	0	0	0
0.5-1.5	0	3	3	0	0	1	2	3	3
0.7-1.3	0	2	2	0	0	0	1	3	2
**Clearance (total) n = 3**									
Ratios	2.52	1.43	0.82	5.25	2.99	1.70	1.79	1.25	0.75
	2.33	1.32	0.75	6.90	3.93	2.23	2.33	1.00	1.22
	3.33	1.89	1.08	7.96	4.53	2.58	1.30	1.42	1.21
<0.5	0	0	0	0	0	0	0	0	0
>2.0	3	0	0	3	3	2	1	0	0
0.5-1.5	0	2	3	0	0	0	1	3	3
0.7-1.3	0	1	3	0	0	0	1	2	3
**Half-life (total) n = 3**									
Ratios	1.15	1.15	1.15	1.15	1.15	1.15	7.01	1.98	3.35
	0.94	0.94	0.94	0.94	0.94	0.94	0.94	0.94	1.22
	0.89	0.89	0.89	0.89	0.89	0.89	1.70	0.93	1.90
<0.5	0	0	0	0	0	0	0	0	0
>2.0	0	0	0	0	0	0	1	0	1
0.5-1.5	3	3	3	3	3	3	1	2	1
0.7-1.3	3	3	3	3	3	3	1	2	1

Table S9 continued

Range	V = 1.0	V = 0.9	V =0.8	V = 1.0 CL = 0.85	V = 0.9 CL = 0.85	V = 0.8 CL = 0.85	V = 1.0 V = 0.8	V = 0.9 V = 0.8	1 species scaling
**AUC (conjugate) n =2**									
	0.36	0.64	1.13	0.12	0.22	0.38	0.36	0.68	1.22
	0.56	0.98	1.72	0.23	0.41	0.72	0.70	1.07	1.56
<0.5	1	0	0	2	2	1	1	2	0
>2.0	0	0	0	0	0	0	0	0	0
0.5-1.5	1	2	1	0	0	1	1	2	1
0.7-1.3	0	1	1	0	0	1	1	1	1
**Clearance (conjugate) n =2**									
Ratios	2.75	1.57	0.89	8.16	4.64	2.64	2.75	1.48	0.81
	1.80	1.02	0.58	4.30	2.45	1.39	1.42	0.93	0.64
<0.5	0	0	0	0	0	0	0	0	0
>2.0	1	0	0	2	2	1	1	0	0
0.5-1.5	0	1	2	0	0	1	1	2	1
0.7-1.3	0	1	1	0	0	0	0	1	1
**Half-life (conjugate) n = 2**									
Ratios	0.75	0.75	0.75	0.75	0.75	0.75	0.75	0.97	1.30
	1.14	1.14	1.14	1.14	1.14	1.14	1.40	1.07	2.31
<0.5	0	0	0	0	0	0	0	0	0
>2.0	0	0	0	0	0	0	0	0	1
0.5-1.5	2	2	2	2	2	2	2	2	1
0.7-1.3	2	2	2	2	2	2	2	2	1
